# Reconstruction and Application of Protein–Protein Interaction Network

**DOI:** 10.3390/ijms17060907

**Published:** 2016-06-08

**Authors:** Tong Hao, Wei Peng, Qian Wang, Bin Wang, Jinsheng Sun

**Affiliations:** 1 Tianjin Key Laboratory of Animal and Plant Resistance/College of Life Sciences, Tianjin Normal University, Tianjin 300387, China; joyht2001@163.com (T.H.); crystal6062@163.com (W.P.); 18222565925@163.com (Q.W.); wbkjc@mail.tjnu.edu.cn (B.W.); 2 Tianjin Aquatic Animal Infectious Disease Control and Prevention Center, Tianjin 300221, China

**Keywords:** protein–protein interaction network, reconstruction technique, interactome, proteome

## Abstract

The protein-protein interaction network (PIN) is a useful tool for systematic investigation of the complex biological activities in the cell. With the increasing interests on the proteome-wide interaction networks, PINs have been reconstructed for many species, including virus, bacteria, plants, animals, and humans. With the development of biological techniques, the reconstruction methods of PIN are further improved. PIN has gradually penetrated many fields in biological research. In this work we systematically reviewed the development of PIN in the past fifteen years, with respect to its reconstruction and application of function annotation, subsystem investigation, evolution analysis, hub protein analysis, and regulation mechanism analysis. Due to the significant role of PIN in the in-depth exploration of biological process mechanisms, PIN will be preferred by more and more researchers for the systematic study of the protein systems in various kinds of organisms.

## 1. Introduction

Protein–protein interaction (PPI) refers to the physical binding of two or more proteins as responses to different disturbances and circumstances, which provide considerable adaptability for biological cells to adapt flexibly to the changing environmental conditions [1]. Based on the PPIs, more systematic protein networks were established gradually, known as the protein-protein interaction network (PIN). As most biological networks, PIN manifest scale-free and small-world properties [2]. Scale-free represents that the connectivity distribution of nodes in a network fits a power law. The scale-free property indicates that a PIN consists of a few highly-connected proteins (hub proteins) and a large amount of less-connected proteins, which makes a network tolerate a random protein removal, but sensitive to the removal of hubs [3]. Small-world indicates that any two nodes in a network can be connected with a small number of links, while the average path length between nodes in PINs is much shorter than a random network due to the existence of hub proteins. PIN is a major component of interactomes, which also include other molecular interactions in the cell, such as genetic interactions [4]. Most commonly, interactome refers to the PIN or its subsets. Furthermore, PIN is an effective tool for understanding the complex world of biological processes inside the cell and solving various biological problems in signal transduction, gene regulation, and metabolism [5]. Given the significant importance of PINs, proteome-wide interaction networks have been studied in many organisms from prokaryote [6] to eukaryote [7], from unicellular [8] to human [9], in the last fifteen years. The technique of collecting protein interaction datasets for reconstructing a PIN is improving and the applications of PINs have spread into more and more areas of the biology research. Moreover, the analysis based on PINs leads to accumulation of massive amounts of data concerning protein interaction pairs, protein complexes, and protein functions. The biological hypotheses deduced from PINs play a major role in guiding scientists to understand further the mechanism in cells and design more reasonable experiments for investigating the mystery of protein systems in various organisms.

In this review, the development of PIN in the last fifteen years is discussed, including the reconstructed PINs for different organisms, techniques for rebuilding a PIN, and applications of PINs on function annotation, subsystem investigation, evolution analysis, hub protein analysis, and regulation mechanism analysis.

## 2. PINs for Various Organisms

**Figure 1:**
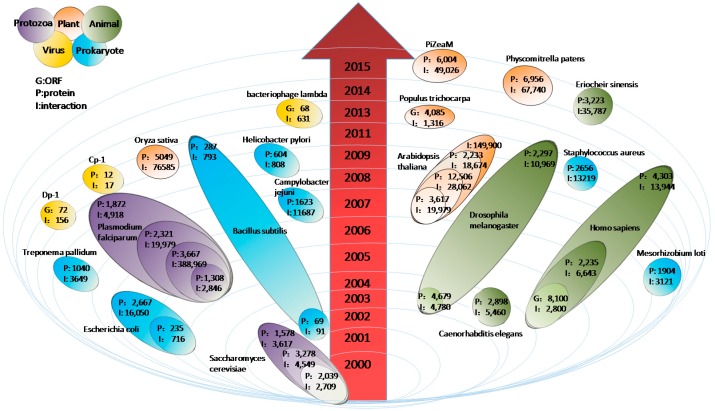
A time chart of the PINs reconstructed in the past fifteen years. The timeline is marked by ellipsoids placed to indicate the year that the PIN was published. The yellow, blue, purple, red, and green ellipsoids represent virus, prokaryote, protozoa, plant, and animal, respectively, for which the PINs have been reconstructed. The letters G, P, and I represent the number of ORFs, proteins, and interactions, respectively, in the indicated version of the PIN. For multiple models of the same species, the numbers of ORFs/proteins and interactions are distinguished by different shades of a color.

The study of PINs has covered a wide range of lifeforms, from viruses to humans, with tens of PINs reconstructed (Figure 1). Systematic analysis of the interactions of massive proteins in a biological system has played significant role on the understanding of the functional principle of proteins and the response of cells to certain special physiological status under diseases or environmental disturbances. Although many organisms have been comprehensively studied for their protein-protein interactions, none of the PINs are capable of capturing all of the interactions in the cell. In fact, for most studies, the proteins detected with interactions usually cover no more than 30% of the whole proteome, which indicates the broader development potentiality of PIN research.

### 2.1. Prokaryote

PINs of prokaryote mostly focus on bacterium, including several model organisms and the pathogens for plants, animals, and humans. For several more concerned organisms, multiple PINs were reconstructed by different research groups with the various approaches for the further investigation of latent information in the cell. For example, the protein–protein interactions of *Escherichia coli*, one of the best studied model organisms, were explored with two different networks [10,11]. Butland *et al.* [10] detected the interactions of 857 *E. coli* proteins and finally obtained 716 stringent interactions involving 83 essential proteins and 152 non-essential proteins. A core sub-network was found broadly conserved across prokaryotes in this PIN, which is composed of 154 interactions and 71 proteins conserved in more than 125 genomes obtained by BLAST homologous searching in different organism genomes. This network provides an available access to the study of protein interaction in prokaryotes. Arifuzzaman *et al.* reconstructed the second PIN of *E. coli* with an analogous method as Butland *et al.* The PIN is composed of 16,050 interactions among 2667 proteins, including 798 uncharacterized proteins [11]. There were 521 common proteins in this network compared with that reconstructed by Butland *et al.* The common proteins were connected by 3088 and 5030 interactions in the two networks, respectively. However, only 218 common interactions were found in those interactions. This indicates that large differences may exist between PINs of the same organism reconstructed from different data sources and experimental strategies. However, both networks show an obvious scale-free property. Additionally, the connectivity of essential genes in Arifuzzaman’s network was consistent with the conclusion that proteins with high essentiality intend to have many interactions [12]. These results, to some extent, support the reliability of the networks.

The PIN of *Bacillus subtilis*, an important industrial bacterium, started from a small network consisting of 91 interactions among 69 proteins [13] and was then extended to include further the interactions focused on several essential cellular processes [14]. The final PIN includes 287 proteins and 793 interactions, which connect the processes of cell division, cell responses to stresses, the bacterial actin-like cytoskeleton, chromosome maintenance, and DNA replication. The reconstructions of PINs for *B. subtilis* prove that the extension of current PIN is an efficient way for network improvement and a larger scale of information mining.

For the pathogens, the detection of differentially-expressed proteins after infection is a specific approach for the reconstruction of a disease- or immune-related PIN. Kim *et al.* [6] filtered the significantly differentially-expressed genes before and after infection with *Helicobacter pylori*, a pathogen that causes various gastroduodenal diseases in animals and humans. With querying to the Uniport database [15], the differentially-expressed genes were converted into proteins and the protein-protein interactions were further investigated based on the human Protein-protein Interaction Prediction (PIPs) database [16] and the Human Protein Reference Database reference (HPRD) [17]. With the integration of these data, a PIN of *H. pylori* infection response was reconstructed, which was composed of 808 interactions and 604 proteins.

Many studies of PINs for prokaryotes focus on the pathogens, such as *Staphylococcus aureus* [18] and *Treponema pallidum* [19], which influence more than one organism in the analysis of a single PIN. Furthermore, the research on the combination of pathogens with human PINs may provide a new platform for human disease studies.

### 2.2. Eukaryote

#### 2.2.1. Protozoa

PINs for eukaryotes are much more numerous than those for prokaryotes. There has been a lot of effort to explore the eukaryotic protein-protein interaction maps through high-throughput methods. However, no global maps have been fully characterized. *Saccharomyces cerevisiae* is the best-characterized organism, with over 90% of its proteins having been screened and the related interactions identified [20,21,22]. Therefore, several PINs of *S. cerevisiae* were reconstructed based on a large number of datasets accumulated in the large-scale identification of protein interactions. Schwikowski *et al.* analyzed 2709 interactions encompassing 2039 proteins in *S. cerevisiae* and diagramed the set of links within a large protein network [8]. They developed a software program based on the graph-drawing library “AGD” to visualize interactions and found that there was only a single large sub-network consisting of 2358 interactions involving 1548 proteins in a total of 204 independent sub-networks. The other sub-networks contained no more than 20 proteins in which 193 networks contained four, or even fewer, proteins. The accuracy of function annotation with PIN is 72% for the proteins with at least one characterized partner. Ito *et al.* [23] built a dataset of 4549 interactions containing 3278 proteins in *S. cerevisiae*. The largest sub-network in the PIN includes the vast majority of the proteins (87%) and interactions (94%). Ho *et al.* [24] detected 3617 interactions among 1578 proteins by high-throughput mass spectrometry and gained a sub-network of DNA damage response and two sub-networks about signaling pathways based on kinase. With the rapid development of the protein interaction network, it is, of course, superior to traditional methods for function annotation and sub-network exploration. Meanwhile, more and more scientists concentrate on the study of PINs, creating a stepping-stone for comprehensive analysis of other organisms.

PINs for pathogens also exist in the eukaryote. Several PINs of *Plasmodium falciparum* were reconstructed based on *in vivo* [25] or *in silico* methods [26,27,28,29]. Some features of the network were analyzed with the PIN reconstructed by LaCount *et al.* [25], including the degree of interconnectivity, mRNA abundance profiles, and enrichment of GO annotation, to extend the number of protein interactions and illustrate the metabolic pathways and invasion process of *P. Falciparum*. The reconstructed computational models have a larger scale but might contain a lot of false positive items [27,28]. To improve the quality of the reconstructed computational models, the experimental data, such as transcriptional profiles, were introduced to refine the PINs [26].

#### 2.2.2. Plants

PINs for plants were mostly reconstructed in the past eight years. As the data obtained from high-throughput technologies in plants is far from depicting the global PPI maps in a plant cell, most PINs for plants are reconstructed with the computational methods, which predict PPIs by sequence alignment [30,31] or integration of various current datasets [32]. Geisler-Lee *et al.* [7] identified 19,979 interactions for 3617 proteins in *Arabidopsis thaliana* by aligning with *S. cerevisiae*, *Caenorhabditis elegans*, *Drosophila melanogaster*, and *Homo sapiens*, including 1159 high-confidence, 5913 medium-confidence, and 12,907 low-confidence interactions. The confidence levels were identified based on three factors: (1) the number of datasets from which the interaction was predicted; (2) the kinds of experiments supporting the interaction; and (3) the number of species where the interaction was found. They found that the interacted proteins tend to locate in the same subcellular location based on the distribution analysis of interaction pairs. This hypothesis supplied a valuable reference for the prediction of protein functions, novel complexes and pathways, as well as providing more information on the known protein complexes and pathways. Cui *et al.* created an interactome of *A. thaliana* and collected the interactions into a database named *Arabidopsis thaliana* Protein Interactome Database (AtPID) [33]. The database contains 28,062 interactions and 12,506 proteins with 23,396 interaction pairs generated from prediction methods, and the rest of the 4666 pairs from the manual revision with literature or enzyme complexes in the KEGG database [34]. AtPID provides a platform for researchers to further study PIN and molecular function in *Arabidopsis*. Moreover, the computational prediction is shown to be a significant complement to *in vivo* experiments in the discovery of novel proteins. Furthermore, De Bodt *et al.* [35] found 51,885 protein-proteins interactions among 3014 proteins in *A. thaliana* by identifying the orthologous groups from *S. cerevisiae*, *C. elegans*, *D. melanogaster*, and *H. sapiens*. With the filter of GO biological process similarity, GO cellular component similarity, and the Pearson correlation coefficient, the confidence of interactions was improved. Ultimately, they obtained a filtered interactome of 18,674 interactions among 2233 proteins. However, less than 15% of the filtered interactions were covered by the PIN reconstructed by Geisler-Lee *et al.* and, in general, a quite small overlap of the filtered interactions with AtPID can be observed, which indicates the quite different results from the use of different interaction databases and different reconstruction techniques. Lin *et al.* [36] provided an *Arabidopsis* PIN inferred from multiple pieces of evidence, such as homologous interactions, annotation, co-expression, co-localization, and co-evolution, presenting a predicted dataset, namely PAIR (Predicted Arabidopsis Interactome Resource). The dataset holds 149,900 potential molecular interactions, which are expected to cover about 24% of the entire interactome with about 44% precision compared with reported experimental interactions [37].

The PIN of *Populus trichocarpa* was built with a genetic algorithm (see Section 3.3.2) [38]. Four PINs for *P. trichocarpa* were built at four different confidence levels, which were collected from the DOMINE database and evaluated according to the number and kind of resources from which the interactions were predicted [39]. The PINs for confidence levels 0.55, 0.65, 0.75, and 0.85 include 481,253 interactions/19,321 genes, 178,232 interactions/14,536 genes, 42,503 interactions/7501 genes, and 4085 interactions/1316 genes, respectively. The biological plausibility of the predicted interactions was evaluated with the similarity of annotations and expression profiles of interactions in the PIN. By increasing the confidence scores, a certain amount of interactions/genes are lost, but the similarity of pathways and molecular functions for the proteins in each interaction pair is augmented. Meanwhile, the co-expression frequency for each interaction pairs also increases, which can be used as a predictor of protein interaction in PAIR [36].

#### 2.2.3. Animals

The PINs for animals mostly focus on the model organism, such as *D. melanogaster* [40,41], *C. elegans* [42], and *H. sapiens* [9,43,44]. Giot *et al.* [40] reconstructed a protein interaction network of *D. melanogaster*, which contains 4780 interactions among 4679 proteins. Two levels of organization were found in the network through statistical analysis: local connectivity and more global connectivity. The former reflected the interactions within protein complexes, and the latter potentially represented the communication between different protein complexes. Another PIN of *D. melanogaster* was reconstructed by Guruharsha *et al.* [41]. They created the large-scale *Drosophila* Protein interaction Map (DPiM) which included 10,969 high-confidence co-complex membership interactions involving 2297 *Drosophila* proteins.

Due to the increased attention to the health problems, human PINs are usually correlated to diseases. Rual *et al.* [9] reconstructed a dataset, CCSB-HI1, for *H. sapiens* containing about 8100 available Gateway-cloned open reading frames and 2800 protein interactions, 78% of which were verified through an independent co-affinity purification assay. The CCSB-HI1 dataset revealed more than 300 novel protein interactions, including 100 disease-related proteins. This research plays a critical role in the human interactome project. Rob *et al.* [43] reconstructed another large-scale *H. sapiens* protein interaction network containing 6643 interactions and 2235 proteins. With in-depth mining of this dataset, they uncovered some novel, previously-unknown, protein interactions and associations between pathways. It is a major step toward studying human disease in the future. The third proteome-scale human interaction network is HI-II-14, which was reconstructed based on literature and validated with different experiments [44]. HI-II-14 is the state-of-the-art largest experimentally-determined interaction map, including 13,944 interactions among 4303 proteins. The analysis of cancer proteins with HI-II-14 indicates that the cancer-associated proteins tend to form sub-networks correlated to tumorigenesis, which shows the capability of HI-II-14 in prioritizing cancer genes on the systematic view.

In addition to the model organisms, certain important economical species have also attracted the interests of some researchers. Hao *et al.* [45] proposed a PIN for *Eriocheir sinensis* eyestalk, Y-organ, and hepatopancreas, based on the transcriptome sequencing and proteomes of six model organisms including *D. melanogaster*, *C. elegans*, *H. sapiens*, *Rattus norvegicus*, *Mus musculus*, and *S. cerevisiae*. It is the first large-scale PIN for an aquatic crustacean. This map was used as an effective tool for the function annotation of proteins, extraction of signal sub-network, and evolutionary analyses.

### 2.3. Virus

Bacteriophages T7, λ, P22, and P2/P4 (from *E. coli*), as well as ϕ29 (from *B. subtilis*), are among the best-studied bacterial viruses. An abundance of work has been done on learning the function of protein-protein interactions in the life cycles of phages [46], especially for the model phage λ, which is the best-studied template phage and has been investigated since the early 1950s. Finally, tens of interactions between phage λ and its host, *E. coli*, have been investigated [47,48]. Rajagopala *et al.* [49] identified 97 interactions based on 68 ORFs in phage λ. They further screened the interaction in phage λ combining with their hosts *E. coli* [50]. Totally 631 interactions were found, leading to a set of 62 high-confidence interactions after multiple rounds of retesting. This map unraveled the novel regulatory interactions occurring between the *E. coli* transcriptional network and λ proteins, which opened a new door for the thorough understanding of biological regulation mechanisms. Recently, the research on pathogen-host interactions attracts many interests with several virus-human PINs reconstructed [51,52,53], which were proved to be effective in the identification of essential molecular components for the infection of the virus to the human immune system [54].

## 3. Major Techniques Used in PIN Reconstruction

### 3.1. Yeast Two-Hybrid (Y2H)

Y2H was first proposed in 1989 [55]. It is a high-throughput method applied in the discovery of protein-protein interactions *in vivo*. The technique is based on the use of transcription factors, such as Gal4, which contains two domains: transcription activation domain (AD) and DNA-binding domain (BD). AD and BD are separated, firstly, with BD fused to the interest protein as bait and AD fused to another protein as prey. The interaction between bait and prey can elicit the reconstitution of AD and BD to function together as a transcription factor, which can direct the expression of the reporter gene downstream [55]. In addition to supplying a data source for reconstructing PINs, Y2H has been widely used in drug discovery [56], the study of plant cell signaling system [57], and the complexity of membrane traffic machinery [58]. The system is low-cost, accessible, easy to operate, and quick to obtain results. Therefore, it is one of the most popular methods in most laboratories. Additionally, because the approach is carried out *in vivo*, it shows the natural environment, to some extent, in the cell. Researchers can detect weak and transient interaction due to the accumulative effect of gene product [1]. Furthermore, the adjustment of bait and prey proteins enable its suitability for different cells; for example, the MAPPIT method, which takes an engineered JAK kinase without a STAT activation site fused to the interest protein as bait and an active STAT binding site fused to another protein as prey, can be used for mammalian cells [59].

Many PINs were reconstructed based on Y2H, including PINs of *S. cerevisiae*, *D. melanogaster*, *E. coli*, *P. Falciparum*, *C. elegans*, *T. pallidum*, *C. jejuni*, and *H. sapiens*, especially for the PINs reconstructed before 2006. Interestingly, the different PINs generated based on Y2H for the same organism usually have quite a low overlap. For example, the PIN of *S. cerevisiae* constructed by Ito *et al.* [23] has only 141 common interactions with the one built by Schwikowski *et al.* [8], which reflects the high false-positive rate of Y2H. The careful follow-up analysis is necessary to identify true, biologically-relevant interactions.

### 3.2. Affinity Purification and Mass Spectrometry 

Mass spectrometry (MS) is usually used coupled with an affinity purification method for the detection of protein interactions, such as affinity purification coupled with mass spectrometry (AP/MS), tandem affinity purification coupled with MS (TAP/MS), and co-affinity purification coupled to mass spectrometry (coAP/MS). Different from the pull-down method, which fixes the “bait” protein indirectly through tags, AP/MS directly immobilizes the “bait” protein on a solid support to capture target proteins from a soluble phase *in vitro*. The captured proteins are then digested into peptides and detected with MS. The bait protein can be endogenous or a protein fused to an “epitope tag”. The limitation of this method is that the result is usually influenced by the proteins co-purified from affinity purification, improper folding and mislocalization [1]. TAP/MS and coAP/MS can overcome these limitations through *in vivo* interactions. TAP/MS is also based on the use of tags attached to the terminus of the target proteins. The genes encoding tags and target proteins are carried by a retrovirus to transfer and express in a host cell. Then, the target protein complexes are isolated by two steps of affinity purification. Coupled with mass spectrometry, the target proteins, and their interactions are identified [60]. coAP/MS uses antibodies instead of tags, compared to TAP/MS, which allows investigation for multiple isoforms and eliminates the influence of protein conformation by tags. Excluding the artificial effect, TAP/MS and coAP/MS detect the protein interactions at the level of most natural conditions. Additionally, the proteins detected with these two methods are post-translationally modified. Through these approaches, many interactions can be detected in one experiment with higher accuracy. The disadvantage of TAP/MS and coAP/MS is that the low-affinity or transient interactions may be missed in the detection. Moreover, it is less sensitive when two proteins interact indirectly through the mediation of third-party proteins. Sometimes, the tag used in TAP/MS will hinder the interactions (false negative). Mostly, we do not know the internal structure of the complex [1]. However, because of their low false positive results and high throughput, they are widely used in the reconstruction of PINs. Additionally, many PPIs generated from other approaches, such as FRET, BERT, and flow cytomery, *etc.*, which have been described in a previous review [1], can also generate non-negligible resources for the reconstruction of PINs.

The *DPiM* [41] was reconstructed based on the coAPMS, which also used in the detection of protein complexes for *S. cerevisiae* [61], *E. coli* [62], and *Mycoplasma pneumonia* [63]. Two of the *E. coli* PINs were reconstructed based on the TAP/MS method by Butland *et al.* [10] and Arifuzzaman *et al.* [11], respectively. The difference is that the latter used a smaller His-tag at N-terminal of *E. coli* ORF rather than the larger SPA tags at the C-terminal of ORFs used in the former work. Furthermore, an overproduction system with multi-copy plasmid clones was used in the work of Arifuzzaman *et al.* to avoid sensitivity problems but, in the mean time, it lost stoichiometry between bait and prey proteins. The scales of the two PINs are quite different, and the overlap is rather low. As the two PINs were reconstructed with different tags and overproduction system, it may indicate that these two factors have a significant influence on the final result, with the different tags may affect the conformation of proteins [64] and the overproduction system may change the strength of protein-protein interactions.

### 3.3. Prediction Based on Computational Method

Although the experimental method has detected thousands of PPIs in various model organisms, the current size of the interactome detected experimentally usually constitutes quite a small part of the whole genome size [65]. Meanwhile, the data from experimental detection still suffers from high rates of false positives and negatives [66]. Moreover, a vast number of PPIs for non-model species are still unclear, which hinders the development of PINs in further investigating for more species [67]. The demand for additional PPIs in more species has led to the development of the computational prediction of PPIs over the past decade.

#### 3.3.1. Interolog-Based Method

Interologs are defined as the protein-protein interactions that are conserved in two species [68]. The interolog-based method is based on the assumption that evolutionarily-conserved proteins tend to have conserved interaction [69,70]. It has been used as a reference for the prediction of a new PIN [71]. This method was used to present the predicted *Arabidopsis* PIN combined with four reference model organisms, *S. cerevisiae*, *C. elegans*, *D. melanogaster*, and *H. sapiens* [7]. The homology search among eukaryote is not rare in the reconstruction of PINs. The reconstruction of *P. Falciparum* also includes the dataset obtained by comparing proteins of *P. Falciparum* with previously-known sequences in other organisms [26,27,28]. The interologs can be searched in the organisms from protozoa, plants, animals, to human, such as the PINs reconstruction of *Arabidopsis* and *E. sinensis*. However, the interolog-based method is incapable of detecting the PPIs with non-conserved proteins. Interestingly, the reconstruction of the *H. pylori* PIN is based on the distant homology search of interologs between bacteria and human [11], which might be because of the close relationship between these two organisms as pathogen and host. The accuracy of the distant search can be controlled by the reference validation, whereas some relevant biological proteins might be missed in such a method, which can be partly complemented by an extensional search regardless of the connectivity of the network.

#### 3.3.2. Prediction Based on Genetic Algorithms

Genetic algorithms are a search heuristic that mimics the process of natural evolution to find an optimum solution to a problem. In recent years, the genetic algorithm has usually been used in the prediction of PPIs based on the features of interacted proteins, such as the residue profiles [72] or domains [73]. A domain-based genetic algorithm, Elucidating Network Topology with Sequence (ENTS), was proposed for the reconstruction of PINs [38]. This approach utilizes the pairwise combinations of conserved domains and predicts subcellular localization of proteins as input features. It has been used to predict the interactions of *A. thaliana*, *P. trichocarpa*, *M. musculus*, *H. sapiens*, and *S. cerevisiae*. The PIN of *A. thaliana* reconstructed by ENTS includes more than twice as many predicted interactions as the PIN reconstructed by Geisler-Lee *et al.* [7] and De Bodt *et al.* [35]. By contrast, ENTS made similar prediction accuracy with AtPID [32] and less accuracy than PAIR [36]. However, the coverage between the PINs from ENTS and the other four datasets are rather poor, with the highest coverage between the ENTS and PAIR dataset at 36.8% of the ENTS predictions.

Interactions predicted by computational methods compensate for the lack of PPIs obtained from experiments to some extent [74]. However, this method is incapable of predicting the interactions between proteins without conserved sequences or detectable interacting domains. The organism-specific proteins and interactions are usually missed in the prediction. The successful combination of the genome-scale network with protein structures and their molecular assemblies [75] provides a novel thought for the prediction and validation of PPIs, which is going to be a trend in the research of PPIs and PINs. Therefore, to some extent, the disadvantage of computational predictions can be compensated by the studies on the structure of interacting proteins, such as protein-protein docking [76]. From this point of view, the protein-protein docking and interacting resources supply different, but important, information of the protein-protein interactions in a wide range of organisms spanning from virus to human. For example, the GWIDD database [77] allows finding interacting proteins for an input sequence/structure and obtaining the structure of the protein complex.

With the accumulation of PPI information obtained from different methods, a series of databases have been established to collect and manage the PPIs or PINs from various organisms. Moreover, the International Molecular Exchange (IMEx) consortium was founded with 16 major public interaction data providers for the purpose of establishing a non-redundant set of protein-protein interactions available in a common website [78]. Protein interactions were carefully checked and a unified file format was developed for representing protein-protein interaction data from different resources, including databases and journals. It provides a new, reliable infrastructure of protein-protein interaction data collection for the researchers working on PPIs and PINs. Table 1 shows the main databases which can be used as data sources for the reconstruction of PINs.

## 4. Application

### 4.1. Function Annotation of Proteins

Although the number of fully-sequenced organisms is increasing and the technique in analyzing the structure of proteins is developing, many proteins remain functionally uncharacterized. Since proteins usually function as complexes, it is hard to find the function of a given protein without knowing what kind of other proteins it interacts with. Function annotation of proteins is a complex and onerous mission by *in vivo* experiment. However, the interaction between an unknown protein with a well-characterized protein can supply a major clue to the function of the former. From this point of view, PINs supply a much more convenient, and relatively reliable way, for the detection of protein functions. Although further verification is still needed for the predicted protein functions, the PINs are of considerable value to predict functions for novel proteins and a step forward to complete our understanding of mechanistic information in proteomes. Here, we elaborated the main methods and cases of protein annotation with PINs in the following sections.

#### 4.1.1. Annotation Based on Adjacency Proteins

As classified and unclassified proteins interact in a vast complex network, PINs naturally serve as a platform to place functionally-unclassified proteins in a biological context, which makes it possible to predict the function of unclassified proteins according to their neighborhood relationship [21]. Schwikowski *et al.* demonstrated that the function and cell localization of interacting proteins have clustering features [8]. Based on the functional similarity, the function of a protein can be predicted as the enriched functions among its interaction neighbors. With this concept, they added annotations to 364 unknown proteins in *S. cerevisiae* by analyzing the function of adjacent proteins and retaining the top three highest frequency function annotations. Alexei *et al.* [95] further took the annotation as an iterative process by considering the function of unclassified proteins annotated in the last turn and proposed a global optimization method. The acceptance or rejection of a function annotation was determined by an optimal algorithm. This method was applied to the PIN of *S. cerevisiae* reconstructed by Schwikowski *et al.* and the accuracy of the prediction was 60%–70% for the proteins with two neighbors. Hishigaki *et al.* [96] extended the one-level neighbor to multiple levels and the number of neighbor levels is determined by a self-consistency test. This method successfully predicts the subcellular localization, the cellular role, and the biochemical function of *S. cerevisiae* proteins with the accuracies of 72.7%, 63.6%, and 52.7%, respectively, based on the ontology annotation from Yeast Proteome Database [97]. Hao *et al.* [45] took the first-level neighbor into consideration and iteratively calculated the annotation of unclassified protein by adopting the top 25% annotations in neighbor proteins. This method avoids the missing of some important annotations, in particular for the proteins with multiple functions, but further validation was needed to filter some spurious annotations. With this method, 549 unclassified proteins were annotated for *E. sinensis*, which made up 76% of all the unknown proteins in the global PIN.

#### 4.1.2. Annotation Based on Cluster Analysis

Modularity is a common property of most networks. The nodes with similar features in a network intend to be classified into the same module/cluster based on different algorithm. For PINs, the proteins located in the same cluster usually reflect their similar function or membership in a complex. Therefore, the modularity feature of PINs has been used in the identification of protein complexes and the function of proteins. Guruharsha *et al.* [41] used DPiM to identify 556 putative complexes encompassing 2240 proteins by the Markov clustering algorithm [98]. This method revealed many known, and hundreds of previously uncharacterized, protein complexes and, thus, provided annotations for 586 proteins that were previously unclassified.

### 4.2. Subsystem Investigation

Investigation of the subsystems by extracting and analyzing the sub-networks is one of the main applications of PINs. Subsystems are easily identified with PIN according to the function or pathway annotation of proteins in the network. A sub-network concerning a specific function is a good platform for deep investigation of the correlated subsystem. Ho *et al.* [24] gained a sub-network of DNA damage response and two sub-networks about signaling pathways based on kinase from a *S. cerevisiae* PIN. Additionally, three sub-networks consisting of proteins involved in autophagy, spindle pole body function, and vesicular transport were also extracted from the global PIN of *S. cerevisiae* [23]. Bryan *et al.* created several sub-networks for different purposes with the PIN of maize [30]. Two highly-conserved sub-networks, which are composed of highly-conserved interactions with interologs in greater than four and five species, respectively, were identified and can be used in the analysis of ancient pathways. A response disease sub-network and several nested sub-networks, such as the MAPK signaling sub-network and *S*-adenosyl methionine synthase sub-network, were also investigated, which can be applied in the analysis of specific responses to pathogens. The signaling transduction sub-network composed of 2039 interactions was extracted from *E. sinensis* and seven classical signaling pathways were found in it [45]. The application on subsystem investigation makes PIN a convenient tool for the studies with a specific biological purpose. Furthermore, the analysis based on a human PIN demonstrated that the cancer related proteins are highly connected in the human interaction network. With the “guilt-by-profiling” concept and “guilt-by-association” predictions, cancer proteins and genes were predicted, including some well-known cancer proteins [44]. The different human diseases also have close connections since the mutant in a single gene may give rise to various disorders. Products of genes that contribute to the same diseases preferred to interact with each other in the PIN. The interaction map of the disorders and disease genes were described as part of diseasome [99]. With the new trend of structure associated interaction studies, dSysMap was developed as a resource for the map of disease-related mutations which link interactions with protein structures and suggest new connections between disorders [100]. The close interactions among proteins for human disorders drive the disease-associated PINs to aid in understanding the phenotype relationships among human diseases and the prediction of disease protein candidates, which provides more unbiased evidence for the research and development of precision medicine.

### 4.3. Evolutionary Analysis

PINs are composed of a vast number of interactions which may have interologs in many other organisms, in particular for the PINs reconstructed based on the interologs. Therefore, PINs can serve as an evolutionary analytical tool for further understanding of the evolution routes of some specific sub-networks [101].

The PIN of *C. elegans* has been used for the evolutionary analysis and shed light on the assumption that new cellular functions rely on the interactions between evolutionarily new and ancient proteins, which is in line with the classical evolutionary theory [42]. Additionally, the analysis of evolution paths for some classical signaling pathways in *E. sinensis* shows that various pathways have different evolution origins with the speculation that Hippo, Jak-STAT, mTOR, and Wnt pathways may grow and mature in the primitive and bilateria stages, respectively [45]. Furthermore, it has been proved that the interacting protein families have similar phylogenetic trees (mirror tree). Therefore, the co-evolution between interacting protein families can also be used to predict PPIs and reconstruct PINs based on the co-evolutionary mirror tree [102].

### 4.4. Hub Protein Analysis

Hub proteins refer to a small, but significant, proportion of proteins which interact with many partners. Biological PINs are particularly robust to the random removal of proteins, but are significantly influenced by the removal of hub proteins. It is demonstrated that the knockouts of hub-related genes in *S. cerevisiae* can lead to the apoptosis with three-fold more possibility compared to non-hub genes [103,104,105]. The essentiality of hubs in a network which has been observed in several model organisms [12,106,107] is commonly referred as the centrality-lethality rule [103]. Han *et al.* revealed two types of hubs, party hubs and date hubs. The former bind most of their neighbors simultaneously, and the latter interact with their different neighbors at different times or locations. In a model with organized modularity, date hubs organize the proteins to connect biological processes or modules to each other, whereas party hubs function inside modules, which has been proved by both *in silico* studies of network connectivity and *in vivo* genetic interactions [108]. However, another view demonstrated that centrality-lethality rule had no business with hubs, but was related to essential PPIs which are indispensable for the survival or reproduction of an organism [109]. In some cases, the deficiency of hubs has an adverse effect on the specific biological function with some essential PPIs involved. Additionally, it is proved that essential PPIs are evolutionarily more conserved than nonessential PPIs. The research on essential PPIs explained the existence of certain essential genes in a molecular view and offered theoretical speculations for future experimental validation. Zotenko *et al.* [110] also demonstrated that most hubs are critical because they are involved in essential complex biological modules, a set of highly connected proteins with common function and abundant essential proteins, which can be recognized by some analytical tools such as ModuleRole [111]. Furthermore, the molecular architecture of protein structures may influence the signal communication among function sites and then affect the interaction among proteins [112], which indicates that the structure and conformation of proteins have an important influence on the topology of PINs. No matter in which opinions, PIN plays a significant role in the analysis of hubs and essential proteins.

In the *H. pylori* PIN [6], with analytical processing aiming at hub and bottleneck proteins, some proteins related to the immune and signal transduction, infection and inflammation, and carcinogenesis were found, which refined the understanding of the processes of inflammation and carcinogenesis and the regulation mechanism within. The process of inflammation and carcinogenesis can be intervened by inhibiting these proteins, which provide an available approach for resolving gastric carcinoma and other cancers. Many groups of highly-connected hubs (GoH) were also found in the PIN of *B. subtilis* [14]. The GoH plays many critical roles in biological function: (1) switching between ‘party hubs’ and ‘date hubs’ can protect important processes from deleterious mutations and would ensure that the relevant processes remain connected in environmental conditions; and (2) the GoH was proved to be associated with the membrane and integrate the external signals. These findings combined with the information of paralogous genes and co-expression genes are helpful for deeply understanding of the highly-redundant functions of the GoH. GoHs can be found through the analysis of cliques in a network, which is a complete subgraph with every two nodes being adjacent [113]. The group connected with different cliques is enriched in dense links, which is just the most important feature of GoH. With the rising generation of the datasets for capturing the response of cells under different disturbs, the static interaction network has been combined with the dynamic expression of proteins to predict the missing expressions from an experiment by contextualizing the network with discrete logic modeling optimization algorithms [114,115]. As GoH functions as a junction in the context of a network, the combination with GoH identification and these computational algorithms might be possible to predict the dynamic cell states in the different intro- or extracellular microenvironment.

### 4.5. Regulation Mechanism Analysis

As interactions in a PIN are not limited in a single organism, the PIN can be a useful analytical tool for the mutual influence between two or more organisms, in particular for the relationship between pathogen/bacteriophage and their hosts. The regulation between phage λ and *E. coli* was analyzed with a phage-host interaction network [50] and 78 shortest paths from 27 genes required for the infection of the phage to proteins targeted by the phage were identified. With the connections from the phage to the required host genes, the transcription factors with an essential role in the regulation of the related genes (such as Crp or Fis [116]) and targeted indirectly by the phage were further identified.

## 5. Conclusions

With the development of high-throughput techniques, PIN attracts more and more interest for the study of interactomes in the model or non-model organisms. The systematic property makes PIN a valuable tool for the large-scale study of interactomes, and the network nature makes it a significant platform for the detailed analysis of certain functions and processes based on the specific sub-networks. In this review, we detailed the published large-scale PINs in the past fifteen years and summarized the related reconstruction methods and applications. The urgent problem in the further development of PINs is to present a cell model which can reflect the true biological process dynamically and precisely. There exist two essential problems in the way to achieve this goal. First, the data from a high-throughput experiment, such as Y2H and TAP/MS, is not quite stable against the disturbances. Although the computational predictions for PPIs are complementary to the *in vivo* experiment to some extent, the quality of the reconstructed PINs is still far from satisfaction. The generation of interactions from protein structures might be a trend to invalidate the existing PPIs or predict new PPIs. Second, the PINs show the static situation of the interactions, whereas PPIs in a cell are time-variant. Therefore, the reconstruction of dynamic PINs is another challenge to further study. With these limitations, much more effort is still needed to make PIN a more efficient tool for the research on interactomes and proteomes in the cell.

## Figures and Tables

**Table 1:** Main databases containing large-scale PPIs.

Database	URL	Number of Interactions *	Number of Species	Data Source *	Ref.
BIND	http://bind.ca	58,266	10	E	[79]
BioGRID	http://thebiogrid.org	1,055,196	30	E	[80]
HGPD	http://www.hgpd.jp/	34,624	human	E	[81]
HPRD	http://www.hprd.org/	38,167	human	E	[17]
PIPs	http://www.compbio.dundee.ac.uk/www-pips	79,441	human	C	[16]
I2D	http://ophid.utoronto.ca/	687,072 (E) 619,398 (C)	6	E; C	[82]
HitPredict	http://hintdb.hgc.jp/htp/	398,696	105	E	[83]
IID	http://ophid.utoronto.ca/iid	1,566,043	6	E; C	[84]
IntAct	http://www.ebi.ac.uk/intact	586,731	275	E	[85]
iRefWeb	http://wodaklab.org/iRefWeb/	263,479	1,448	E	[86]
MINT	http://mint.bio.uniroma2.it/mint/	235,635	~30	E	[87]
PAIR	http://www.cls.zju.edu.cn/pair/	5990 (E) 145,494 (C)	*A. thaliana*	C, E	[36]
PINA	http://cbg.garvan.unsw.edu.au/pina/	365,930	6	E	[88]
AtPID	http://atpid.biosino.org/	28,062	*A. thaliana*	C	[33]
STRING	http://string-db.org	919,186,040	2,031	C	[89]
DIP	http://dip.doembi.ucla.edu	81,067	808	E	[90]
MatrixDB	http://matrixdb.ibcp.fr	9,851	Human	E	[91]
InnateDB	http://www.innatedb.com	227,297 (E) 248,205 (C)	Human Bovine Mouse	C, E	[92]
HPIDB	http://www.agbase.msstate.edu/hpi/main.html	45,482	70 host and 598 pathogens	C, E	[93]
Interactome 3D	http://interactome3d.irbbarcelona.org/	12,000	8	C	[94]

* C: Computational prediction; E: Experimental detection.
